# Modulation of Cell Surface Receptor Expression by Modified Vaccinia Virus Ankara in Leukocytes of Healthy and HIV-Infected Individuals

**DOI:** 10.3389/fimmu.2020.02096

**Published:** 2020-09-08

**Authors:** Adrien Leite Pereira, Quentin Jouhault, Ernesto Marcos Lopez, Antonio Cosma, Olivier Lambotte, Roger Le Grand, Michael H. Lehmann, Nicolas Tchitchek

**Affiliations:** ^1^INSERM U1184, Immunology of Viral Infections and Autoimmune Diseases, IDMIT Infrastructure, CEA–Université Paris Sud 11, Fontenay-aux-Roses, France; ^2^INSERM U1184, Center for Immunology of Viral Infections and Autoimmune Diseases, Le Kremlin-Bicêtre, France; ^3^APHP, Service de Médecine Interne et Immunologie Clinique, Hôpitaux Universitaires Paris Saclay, Le Kremlin-Bicêtre, France; ^4^Institute for Infectious Diseases and Zoonoses, Ludwig-Maximilians-Universität München, Munich, Germany

**Keywords:** AIDS, chemokine, cytokine, mass cytometry, modified vaccinia virus Ankara, poxvirus, surface marker, vaccination

## Abstract

Viral vectors are increasingly used as delivery means to induce a specific immunity in humans and animals. However, they also impact the immune system, and it depends on the given context whether this is beneficial or not. The attenuated vaccinia virus strain modified vaccinia virus Ankara (MVA) has been used as a viral vector in clinical studies intended to treat and prevent cancer and infectious diseases. The adjuvant property of MVA is thought to be due to its capability to stimulate innate immunity. Here, we confirmed that MVA induces interleukin-8 (IL-8), and this chemokine was upregulated significantly more in monocytes and HLA-DR^bright^ dendritic cells (DCs) of HIV-infected patients on combined antiretroviral therapy (ART) than in cells of healthy persons. The effect of MVA on cell surface receptors is mostly unknown. Using mass cytometry profiling, we investigated the expression of 17 cell surface receptors in leukocytes after *ex vivo* infection of human whole-blood samples with MVA. We found that MVA downregulates most of the characteristic cell surface markers in particular types of leukocytes. In contrast, C-X-C motif chemokine receptor 4 (CXCR4) was significantly upregulated in each leukocyte type of healthy persons. Additionally, we detected a relative higher cell surface expression of the HIV-1 co-receptors C-C motif chemokine receptor 5 (CCR5) and CXCR4 in leukocytes of HIV-ART patients than in healthy persons. Importantly, we showed that MVA infection significantly downregulated CCR5 in CD4+ T cells, CD8+ T cells, B cells, and three different DC populations. CD86, a costimulatory molecule for T cells, was significantly upregulated in HLA-DR^bright^ DCs after MVA infection of whole blood from HIV-ART patients. However, MVA was unable to downregulate cell surface expression of CD11b and CD32 in monocytes and neutrophils of HIV-ART patients to the same extent as in monocytes and neutrophils of healthy persons. In summary, MVA modulates the expression of many different kinds of cell surface receptors in leukocytes, which can vary in cells originating from persons previously infected with other pathogens.

## Introduction

Protection of humans against infectious diseases by vaccination is considered as one of the greatest successes in the history of medicine. In particular, 40 years ago, the world health assembly officially declared smallpox eradicated. Vaccinia virus (VACV) has been successfully used to vaccinate against smallpox, but it can cause severe side effects (1; 2). Therefore, as an effort to attenuate VACV in a way that increases its safety while keeping its immunogenic potential, chorioallantois vaccinia virus Ankara (CVA) was passaged multiple times in chicken embryo fibroblasts (CEFs). This yielded a modified VACV strain, which does not replicate in primary human cells (3). Vaccination of persons with the modified vaccinia virus Ankara (MVA) was well tolerated in more than 120,000 persons (4), and recently, the safety and efficacy of MVA were confirmed in a phase 3 clinical trial designed for the usage of MVA against smallpox (5).

Additionally, MVA has been widely used as a viral vector in clinical studies intended to treat and prevent cancer and infectious diseases (6–9). The safety of virus-vectored vaccines is intensively discussed and regulatory guidelines for their usage are being established (10; 11). The effectiveness of a vaccine depends not only on its specific composition but also on the individual immunological status of a person to be vaccinated (12). The latter point is highly relevant for the development of therapeutic HIV vaccines because HIV-1-infected patients suffer from chronic inflammation even when receiving antiretroviral therapy (ART) (13).

MVA has been applied as a viral vector in several clinical trials that enrolled HIV-1-infected patients (14–18) and healthy persons (19–21). Therein, MVA has indeed proved to be safe, and its ability to stimulate innate immunity has been considered as a beneficial adjuvant effect (22; 23). Although there are some studies about MVA-induced cytokine expression (24–26), only limited information is available about the effect of this virus on cell surface receptors in leukocytes (27–29). However, such information is necessary for a better understanding of the complex immune responses triggered by this virus in vaccinated individuals.

Moreover, it is necessary to reveal potential differences in vaccine responsiveness between infected and healthy persons to improve vaccine design for example for people living with HIV. Therefore, we investigated by mass cytometry the effects of MVA on cytokine expression and the expression levels of some selected cell surface receptors including C-C motif chemokine receptor 5 (CCR5) and C-X-C motif chemokine receptor 4 (CXCR4), the two major co-receptors for HIV entry (30), in leukocytes of HIV-1-infected patients receiving ART in comparison to healthy persons.

## Materials and Methods

### Patients

Whole-blood samples from five HIV-infected patients on combined ART (HIV-ART patients) and five healthy persons were collected in lithium heparin tubes by the Etablissement Français du Sang (EFS, Hôpital Saint Louis, Paris, France) and the Hôpital du Kremlin Bicêtre, respectively. The age (range), infection route, number of CD4^+^ T cells, viral load, year of HIV detection, year of the beginning of ART, type of ART, and adherence to ART were provided for each HIV-infected patient (Table 1). Their age ranged from 45 to 60 years, the CD4 cell counts from 427 to 811 cells/mm^3^, and the plasma HIV RNA levels were <40 copies/ml. The age (range) of each healthy subject is provided in Table 2.

**TABLE 1:** Characteristics of HIV-ART patients.

Patients	PAT-1	PAT-2	PAT-3	PAT-4	PAT-5
Current age	45–50	55–60	50–55	50–55	55–60
Infection routes	Sexual	Sexual	Unknown	Unknown	Sexual
Number of CD4^+^ T cells (cells/mm^3^)	559	427	624	758	811
Viral load	<40	<40	<40	<40	<40
Detection	2000	1985	2009	1999	1995
Treatments starting	2015	1990	2009	1999	1995
Treatment	Emtricitabine Rilpivirine Tenofovir	Emtricitabine Rilpivirine Tenofovir	Emtricitabine Rilpivirine Tenofovir	Abacavir Lamivudine Dolutegravir	Emtricitabine Disoproxil fumarate Tenofovir
Adherence to treatment	Yes	Yes	Yes	Yes	Yes

*The current age (range), contamination pathway, viral load, year of detection and starting treatment, and ongoing treatment are shown for each HIV-infected patient.*

**TABLE 2:** Characteristics of healthy persons.

Patients	HEA-1	HEA-2	HEA-3	HEA-4	HEA-5
Current age	45–50	60–65	25–30	30–35	55–60

*The age (range) of each healthy subject is shown.*

In this study, viral loads were used to determine whether ARTs were effective. We concluded that the adherence to treatments was correct and the treatments were effective, as the viral loads were <40 copies/ml for all patients.

### Virus

MVA clonal isolate F6 was made available to the CEA by Gerd Sutter (LMU Munich, Germany) on the basis of a Material Transfer Agreement with the Ludwig-Maximilians-Universität München (LMU-MTA). MVA was propagated in primary CEFs, which were cultivated in Eagle’s minimum essential medium (Sigma-Aldrich) supplemented with 2% fetal calf serum. Afterwards, cells were freeze-thawed three times and the cell debris were removed by centrifugation at a relative centrifugal field (RCF) of 453 × *g* for 15 min. The supernatant was centrifuged again at an average RCF of 22,700 × *g* for 3 h. The resulting pellet was dissolved in 10 mM Tris–HCl, pH 9.0, and stored at −80°C. Titration was performed on CEFs as described (31). MVA preparations were regularly screened for potential mycoplasma and other bacterial contaminations.

### Cell Infection, Stimulation, and Storage

Fresh whole-blood samples were infected with MVA at a multiplicity of infection (MOI) of one and incubated at 37°C under 5% CO_2_ in six-well plates (BD Biosciences). After 1 h, brefeldin A (BFA), dissolved in dimethyl sulfoxide (Sigma-Aldrich), was added to the cells at a final concentration of 1 μg/ml to perform intracellular cytokine staining as described (32), and cell incubation was continued for 16 h. Then, cells were fixed and erythrocytes were lysed as described previously (33). In detail, the fixation mixture (FM) contained 18.5% glycerol (Sigma-Aldrich, Lyon, France) in 1X Dulbecco’s phosphate-buffered saline (DPBS) without CaCl_2_ or MgCl_2_, pH 7.4 (Gibco by Life Technologies, Villebon-sur-Yvette, France) and 5% formaldehyde, which was prepared from a 36% paraformaldehyde solution (VWR BDH Prolabo, Fontenay-sous-Bois). Ten-milliliter FM was added to 1 ml blood, which was incubated for 10 min at 4°C and then centrifuged at 800 × *g* for 5 min at room temperature (RT). Red blood cells present in the pellets were lysed by adding 10 ml Milli-Q water. After incubation at RT for 20 min, cells were washed two times with 1X DPBS and centrifuged between washes at 800 × *g* for 5 min at RT. Then, cells were counted, resuspended in FM to 200-μl aliquots containing 3 × 10^6^ cells, and stored at −80°C. This procedure enabled freezing and recovery of all blood leukocytes without damage, especially polymorphonuclear cells, which are highly labile and cryopreservation-sensitive (34).

### Staining and Acquisition

For each sample, 3 × 10^6^ cryopreserved fixed cells were washed twice with staining buffer [PBS-0.5% bovine serum albumin (BSA), Sigma-Aldrich] and labeled with conjugated antibodies according to the following procedures. Cells were incubated at 4°C for 30 min with a mixture of the metal-labeled surface antibodies (Abs) in staining buffer. After two washes with 1X DPBS, cells were incubated in fixation solution [PBS-1.6% paraformaldehyde (PFA), Electron Microscopy Sciences Hartfield] at RT for 20 min, and permeabilized with 1X Perm/Wash buffer (BD Biosciences) at RT for 10 min. Staining with metal-labeled intracellular Abs and an iridium nucleic acid intercalator in 1X Perm/Wash buffer was carried out as for extracellular staining. Cells were stored overnight in 0.1 μM iridium nucleic acid intercalator in a fixation solution. The following day, cells were washed with Milli-Q water, resuspended in 1 ml Milli-Q water, and filtered using a 35-μm nylon mesh cell strainer (BD Biosciences), before the addition of EQ Four-Element Calibration Beads (Fluidigm), according to the manufacturer’s instructions. The acquisition of each sample was manually performed two times in succession on a CyTOF-1 instrument (Fluidigm). The metal and clones of all antibodies used in the mass cytometry panel are shown in Table 3.

**TABLE 3:** Mass cytometry panel.

Metal	Antibody	Clone	Provider
Pr141	CD66	TET2	Miltenyi
Nd142	HLA-DR	L243 (G46-6)	Biolegend
Nd143	CD3	UCHT1	BD Bioscience
Nd144	CD64	10.1.1	BD Bioscience
Nd145	CD86	2331 (FUN-1)	BD Bioscience
Nd146	IL-6	MQ2-13A5	Miltenyi
Sm147	IFN-α	LT27:295	Miltenyi
Nd148	IL-1β	H1b-98	Biolegend
Sm149	CD14	M5E2	BD Bioscience
Nd150	CD11b	ICRF44	BD Bioscience
Eu151	CD38	AT1	Clinisciences
Sm152	CD16	B73.1	BD Bioscience
Eu153	CD154	TRAP1	BD Bioscience
Sm154	CD8A	37006	R&D systems
Gd155	CD32	2E1	Miltenyi
Gd156	CCL4	D21-1351	BD Bioscience
Gd158	IP10	6D4	Clinisciences
Tb159	TNF-α	MAb11	BD Bioscience
Gd160	IL-1α	364/3B3-14	eBioscience
Dy161	NKp80	4A4D10	Miltenyi
Dy162	IL-12	C8.6	Miltenyi
Dy163	Perforin	dG9-DTAG9	BD Bioscience
Dy164	CXCR4	12G5	BD Bioscience
Ho165	CD11a	HI111	BD Bioscience
Er166	CCR5	3A9	BD Bioscience
Er167	IL-8	NAPII	eBioscience
Er168	CD11c	B-ly6	BD Bioscience
Tm169	CD4	L200	BD Bioscience
Er170	CCL5	2D5	BD Bioscience
Yb171	IFN-g	25723	R&D systems
Yb172	CD25	BC96	Biolegend
Yb173	CD123	7G3	BD Bioscience
Yb174	CD19	HIB19	BD Bioscience
Yb175	IL-1RA	AS17	BD Bioscience
Yb176	CCL2	5D3-F7	Biolegend
Ir191/Ir193	Intercalator-Ir	–	–

*The antibodies used in this study are shown along with the metal isotope and clone for each. CCL, C-C motif chemokine ligand; CCR, C-C motif chemokine receptor; CXCR, C-X-C motif chemokine receptor; IFN, interferon; IL, interleukin; TNF, tumor necrosis factor.*

### Characterization of Modified Vaccinia Virus Ankara-Specific Immune Responses

Following data acquisition, cells were gated to exclude beads, doublets, and non-specific background (Supplementary Figure S1A). A Spanning-tree Progression Analysis of Density-normalized Events (SPADE) was performed on the cytometric profiles of the entire dataset (35). The SPADE analysis was parameterized to generate 100 cell clusters using a downsampling of 5%. SPADE clustering was based on the levels of CD3, CD4, CD8, CD11c, CD14, CD16, CD19, CD32, CD64, CD66, CD123, HLA-DR, NKp80, and Perforin (Supplementary Figures S1B,C).

T cell, B cell, natural killer (NK) cell, polymorphonuclear neutrophil (PMN), basophil, monocyte, conventional DC (HLA-DR^high^ and HLA-DR^bright^), and plasmacytoid DC (pDC) populations were identified by annotating clusters generated by the SPADE analysis based on their expression of CD3, CD11c, CD14, CD16, CD19, CD64, CD66, CD123, and HLA-DR (Supplementary Figures S1B,C). HLA-DR^high^ and HLA-DR^bright^ DC populations were defined based on the expression of HLA-DR (Supplementary Figure S1D). Finally, CD4^+^ T and CD8^+^ T-cell populations were split using classical gating (Supplementary Figure S1E).

### Gating Strategy

T cells were identified as CD3^+^, B cells as HLADR^+^ CD19^+^, NK cells as HLA-DR^–^ CD16^+^, PMNs as CD66^+^, basophils as HLA-DR^–^ CD123^+^, monocytes as HLADR^+^ CD14^+^ CD64^+^, conventional DCs as HLA-DR^+^ CD11c^+^ CD64^–^, and plasmacytoid DCs as HLADR^+^ CD123^+^ CD11c^–^. The percentages of each leukocyte population isolated from healthy persons and HIV-ART patients are illustrated in Supplementary Figure S2.

### Cytometry Data Analysis and Statistics

Cytometry data were normalized using Rachel Finck’s MATLAB normalizer based on EQ Four-Element Calibration Beads (36). FCS files were concatenated using the FCS file concatenation tool (Cytobank). SPADE analysis was performed on the Cytobank platform, whereas FlowJo software (TreeStar version 9.9) was used to determine the median signal intensity (MSI) of cell surface receptors (for each cell population) and the percentage number of cells producing cytokines. Phenotypic heatmaps were obtained using Tableau software. Statistical comparisons of cell cluster abundances were performed using the Mann–Whitney test available in R software (R Core Team).

## Results

### Modified Vaccinia Virus Ankara Induces a Higher Percentage Number of Leukocytes Producing Interleukin-8 in the Blood of HIV-ART Patients

It is well established that MVA induces cytokine production (24–26), but it is mostly unknown whether cells of immunocompromised persons including those of HV-1-infected patients respond to this viral vector equally. Therefore, we investigated whether MVA differentially induces cytokine expression in leukocytes of HIV-ART patients and healthy persons. For that, the expression of C-C motif chemokine ligand (CCL)2, CCL4, interferon (IFN)-α, IL-1α, IL-1β, IL-1RA, IL-6, IL-8, IL-12, and tumor necrosis factor (TNF) was determined for each cell type as classified in Supplementary Figure S1.

**FIGURE 1:**
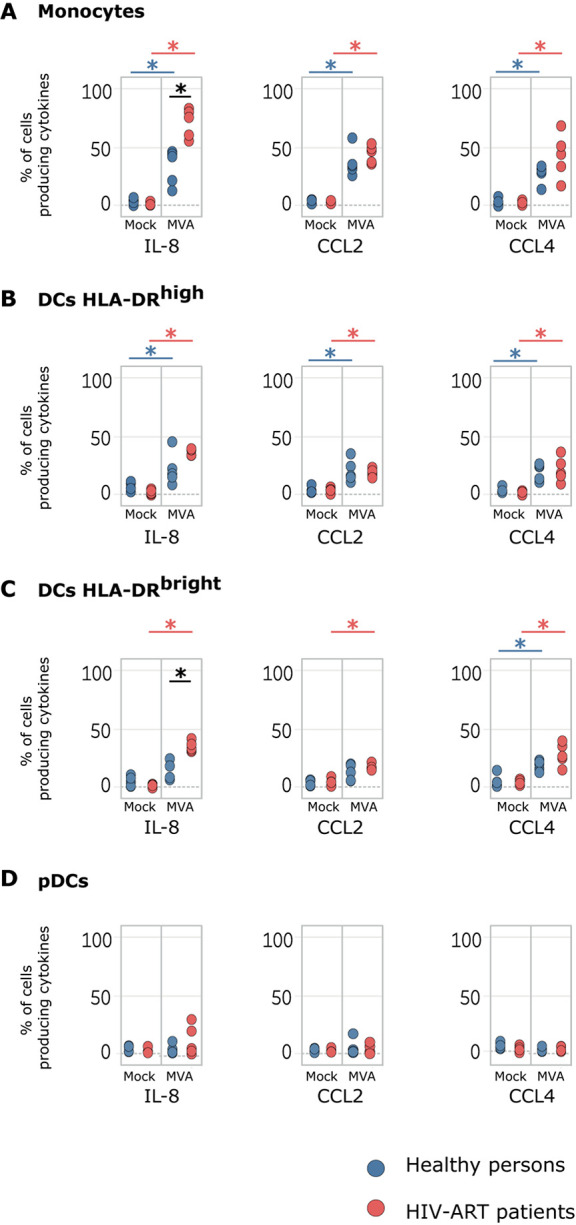
Modified vaccinia virus Ankara (MVA) induces chemokine expression in cells of whole blood from healthy persons and HIV-ART patients. Whole-blood samples of healthy persons and HIV-ART patients were infected with MVA for 16 h or left non-infected. Phosphate-buffered saline (PBS) served as a control. Monocytes, HLADR^high^ dendritic cell (DC), HLADR^bright^ DC, and plasmacytoid DC (pDC) populations were computationally isolated. The percentage numbers of **(A)** monocytes, **(B)** HLADR^high^ DCs, **(C)** HLADR^bright^ DCs, and **(D)** pDCs producing interleukin (IL)-8, C-C motif chemokine ligand (CCL)2, and CCL4 are presented. Blue points correspond to the percentage number of cells obtained from healthy persons; red points to those from HIV-ART patients. Significant differences (*p* < 0.05) between samples are indicated by an asterisk.

We found that *ex vivo* infection of whole blood with MVA significantly induced the production of IL-8, CCL2, and CCL4 in monocytes and HLA-DR^high^ DCs of HIV-ART patients and healthy persons (Figures 1A,B). The percentage number of IL-8-producing monocytes was significantly higher in MVA-infected blood of HIV-ART patients (67.32%) than in MVA-infected blood of healthy persons (31.16%) (Figure 1A). MVA also significantly induced the production of CCL4 in HLA-DR^bright^ DCs of HIV-ART patients and healthy persons, but IL-8 and CCL2 were significantly induced only in HLA-DR^bright^ DCs from MVA-infected blood of HIV-ART patients. Thus, the percentage number of HLA-DR^bright^ DCs producing IL-8 was significantly higher in MVA-infected blood of HIV-ART patients (34.30%) than in MVA-infected blood of healthy persons (20.82%) (Figure 1C). No production of cytokines was detected in pDCs (Figure 1D) and also not in other cell types.

### Modified Vaccinia Virus Ankara Downregulates Cell Surface Markers but Upregulates C-X-C Motif Chemokine Receptor 4 in Leukocytes of Healthy Persons

**FIGURE 2:**
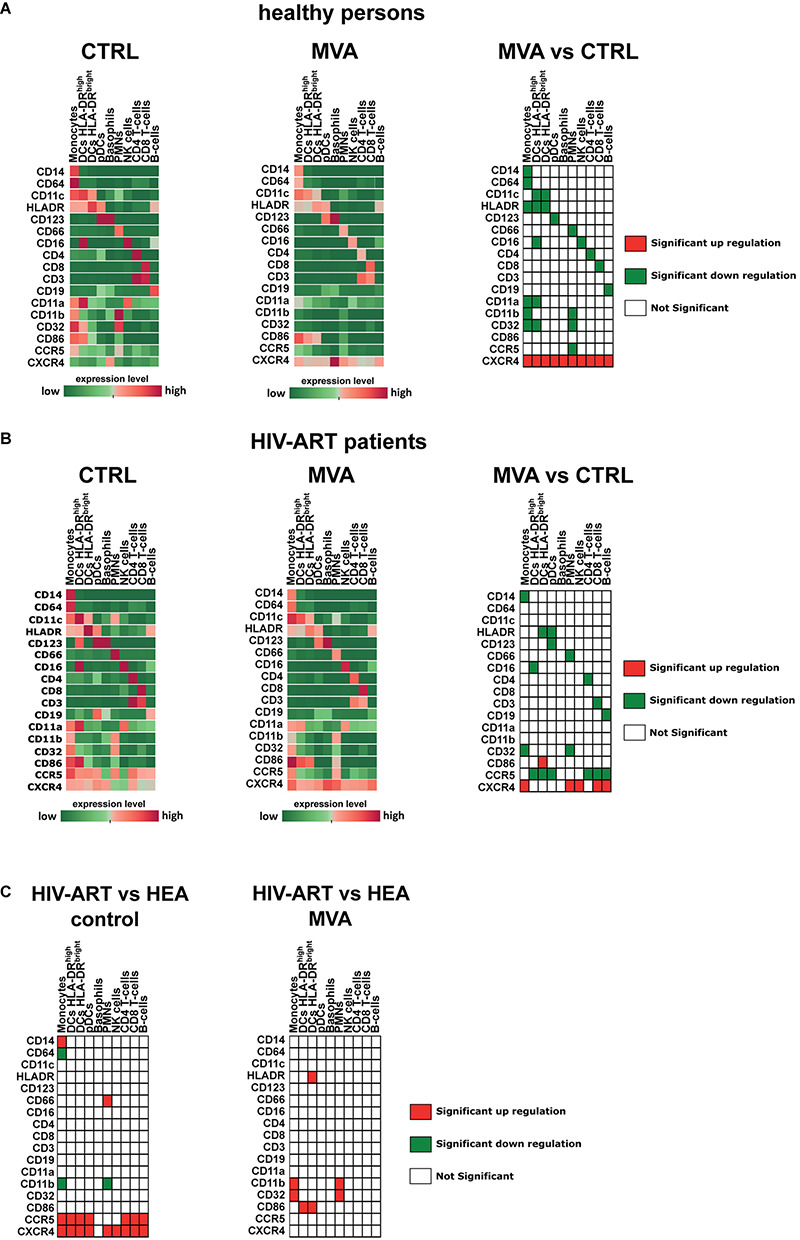
Modulation of cell surface receptor expression by modified vaccinia virus Ankara (MVA) in healthy persons and HIV-ART patients. Whole-blood samples of healthy persons **(A)** and HIV-ART patients **(B)** were infected with MVA for 16 h or left non-infected. Cells were stained with a panel of 35 cell markers and analyzed with Tableau and R software as described in the section “Materials and Methods.” The median expression level [median signal intensity (MSI)] of each cell surface receptor of each cell population is illustrated by an individual heatmap. The expression level ranges from dark green (lowest expression) to dark red (highest expression). Significant differences in the level of cell surface receptor expression between non-infected [**(A,B)** left panel] and MVA-infected [**(A,B)** middle panel] cell populations are indicated for healthy persons [**(A)**, right table] and HIV-ART patients [**(B)**, right table]. **(C)** Significant differences in the level of cell surface receptor expression between healthy persons and HIV-ART patients are indicated for non-infected [**(C)**, left table] and MVA-infected [**(C)**, right table] cell populations. Differences with *p* < 0.05 were considered significant.

**FIGURE 3:**
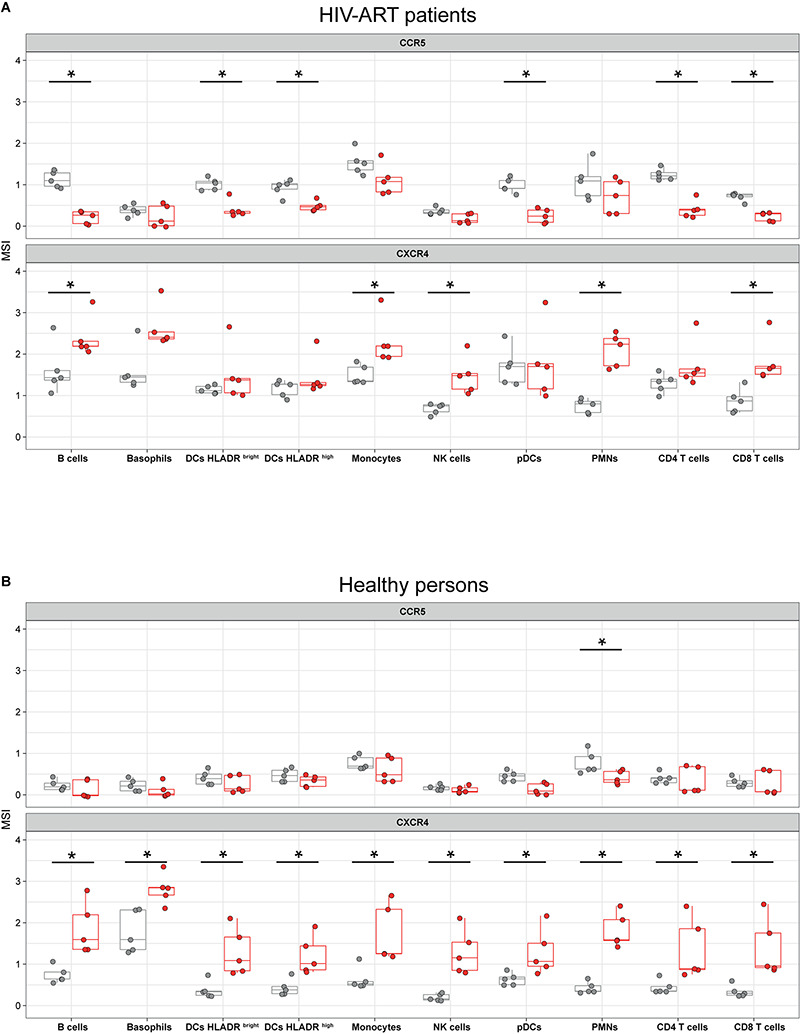
Modified vaccinia virus Ankara (MVA) affects cell surface expression of C-C motif chemokine receptor (CCR5) and C-X-C motif chemokine receptor 4 (CXCR4) in leukocytes of HIV-ART patients and healthy persons. Whole-blood samples of HIV-ART patients **(A)** and healthy persons **(B)** were infected with MVA (red boxes) for 16 h or left non-infected (gray boxes). Cells were stained with a panel of 35 cell markers and analyzed with R software as described in the section “Materials and Methods.” CCR5 and CXCR4 expression levels [median signal intensity (MSI)] of each sample are blotted in the respective jitter plot representations. Significant differences (*p* < 0.05) between samples are indicated by an asterisk.

Studies about the effect of MVA on the expression of cell surface markers are rare (27; 28). However, such information would be very valuable to better understand the intrinsic adjuvant properties of MVA when used as a viral vector in vaccine development (8; 22). Therefore, using mass cytometry, we simultaneously investigated the expression of 17 characteristic cell surface markers (CD3, CD4, CD8, CD11a, CD11b, CD11c, CD14, CD16, CD19, CD32, CD64, CD66, CD86, CD123, CCR5, CXCR4, and HLA-DR) in leukocytes of healthy persons and HIV-ART patients after *ex vivo* infection of whole blood with MVA (Figure 2). MVA downregulated most of the characteristic cell surface markers expressed in the different leukocyte cell types from healthy persons, except for CXCR4, which was upregulated in each cell type investigated (Figures 2A, 3B).

### Chronic HIV-1 Infection Impacts the Expression of Cell Surface Receptors

Previously, it was shown that leukocytes of HIV-infected patients display increased cell surface levels of CCR5 (37; 38). Here, we confirmed by using mass cytometry that CCR5 is significantly upregulated in monocytes, CD4^+^ T cells, CD8^+^ T cells, and B cells of HIV-ART patients as compared with cells of healthy persons (Figures 2A–C, left panels). We also found high CCR5 cell surface expression in all three DC populations but not in natural killer cells. Monocytes of HIV-ART patients additionally had increased cell surface expression of CD14 and decreased cell surface expression of CD64 and CD11b.

CXCR4 cell surface expression was upregulated in each leukocyte type of HIV-ART patients except in basophils, which had the same level as observed in samples of healthy persons. Neutrophils of HIV-ART patients additionally had increased cell surface expression of CD66 and decreased cell surface expression of CD11b (Figure 2C, left panel).

### Modified Vaccinia Virus Ankara Differentially Affects Cell Surface Expression of C-C Motif Chemokine Receptor 5 and C-X-C Motif Chemokine Receptor 4

Since MVA is applied as a viral vector in several clinical trials that enrolled HIV-infected patients (14–17), knowledge about the potential effects of MVA on HIV/AIDS progression would be of great interest. Here we found that infection of whole blood of HIV-ART patients with MVA significantly downregulated CCR5 in B cells (*p* = 0.001), HLA-DR^bright^ DCs (*p* = 0.012), HLA-DR^high^ DCs (*p* = 0.016), pDCs (*p* = 0.008), CD4^+^ T cells (*p* = 0.008), and CD8^+^ T cells (*p* = 0.008) (Figure 3A). In healthy persons, CCR5 expression was significantly downregulated by MVA only in neutrophils (Figure 3B).

In contrast, MVA significantly increased cell surface expression of CXCR4 in B cells (*p* = 0.015), monocytes (*p* = 0.012), NK cells (*p* = 0.008), neutrophils (*p* = 0.008), and CD8^+^ T cells (*p* = 0.008) of HIV-ART patients (Figure 3A) and in each leukocyte population of healthy persons (Figure 3B).

### Modified Vaccinia Virus Ankara Differentially Affects Cell Surface Expression of CD86, CD32, and CD11b

Previously, it was shown in human monocyte-derived DCs that MVA increases cell surface expression of CD86/B7-2 (27), a co-stimulatory molecule for T-cell activation expressed by antigen-presenting cells (39). Here, we observed a slight upregulation of CD86 in HLA-DR^bright^ DCs from MVA-infected blood samples of healthy persons, but this was not statistically different from non-infected cells of healthy persons (Figure 2A). However, CD86 cell surface expression was significantly upregulated by MVA in HLA-DR^bright^ DCs of HIV-ART patients as compared to non-infected cells of HIV-ART patients (Figure 2B). We also observed higher levels of CD86 in monocytes from MVA-infected blood samples of HIV-ART patients as compared to non-infected cells of HIV-ART patients, but, most probably due to the low number of samples, the difference was not significant.

CD32 cell surface expression was significantly downregulated in monocytes and neutrophils of healthy persons (Figure 2A) and HIV-ART patients (Figure 2B) after infection of whole blood with MVA. However, the decrease of CD32 was less in monocytes and neutrophils from MVA-infected blood samples of HIV-ART patients, resulting in significantly higher CD32 levels as compared with cells from MVA-infected blood samples of healthy persons (Figure 2C, right panel).

Modified vaccinia virus Ankara abrogated CD11b surface expression in cells of healthy persons (Figure 2A) but had no significant effect on CD11b surface expression in monocytes and neutrophils of HIV-ART patients (Figure 2B). This resulted in significantly higher surface expression of CD11b in monocytes and neutrophils from MVA-infected blood samples of HIV-ART patients as compared to cells from MVA-infected blood samples of healthy persons (Figure 2C, right panel).

## Discussion

Cell surface receptors enable intercellular communication and thereby they regulate cell proliferation, differentiation, migration, and death. Additionally, cell surface receptors mediate intracellular signaling leading to gene expression and the exchange of molecules with the cell environment. Obligate intracellular pathogens such as viruses can use cell surface molecules to get access to necessary resources, which enable their replication. It depends on the nature of the virus how this works in detail and whether this process is highly specific as in HIV-1 or promiscuous as in orthopoxviruses (40; 41).

In the present study, we confirmed previous findings that CCR5 expression is low in leukocytes of healthy persons and significantly increased in HIV-1-infected patients (37; 38). CCR5 is the main co-receptor of HIV-1 and expressed in many different hematopoietic and non-hematopoietic cell types. Decreased CCR5 surface expression delays AIDS progression and can prevent infection of cells with an R5-tropic HIV strain. It seems even possible to cure HIV-infected patients by transplantation of stem cells having a homozygous CCR5 gene with a 32-bp deletion that causes the total absence of CCR5 at the cell surface (42; 43). Consequently, CCR5 has been recognized as a key drug target against HIV (44), and here we discovered that infection of whole-blood samples with MVA downregulates cell surface expression of CCR5 in DCs, CD4^+^ T cells, CD8^+^ T cells, and B cells of HIV-1-infected patients. This result is in agreement with the finding of Guerra et al. (45) who detected by an RNA microarray that MVA downregulates CCR5 mRNA levels in human monocyte-derived DCs.

Modified vaccinia virus Ankara-infected cells were found in the blood and draining lymph node of cynomolgus macaques, which were inoculated intramuscularly with MVA (46). Systemic spread of MVA was also detected in mice and ferrets although MVA is unable to replicate in most mammalian cells (47–50), suggesting that MVA-infected cells acquire the ability to migrate to lymph nodes and other locations distant from the site of inoculation. Here, we found by mass cytometry that MVA increases cell surface expression of CXCR4 in each cell type of healthy persons but does not further increase the high level of CXCR4 surface expression in DCs, basophils, and CD4^+^ T cells of HIV-ART patients. Thus, MVA-infected cells should migrate into organs, which express C-X-C motif chemokine ligand 12 (51), the only natural agonist for CXCR4 (52). Indeed, this was shown previously for neutrophils, which migrated into the draining lymph node and bone marrow after being infected with MVA in the skin (53). CXCR4 surface expression in leukocytes of HIV-ART patients was increased, which is in accordance with a recent study (54) but in contrast to previous findings in HIV-1-infected patients (38) and simian immunodeficiency virus (SIV)-infected cynomolgus macaques (55).

On the other hand, we could not confirm the upregulation of CD11b, CD32, and CD64 surface expression in monocytes and neutrophils of HIV-ART patients as reported (54). We even detected less CD11b surface expression in monocytes and neutrophils of HIV-ART patients as compared to cells of healthy persons. Upregulation of CD11b surface expression in human monocytes and neutrophils was reported for the reverse transcriptase inhibitor abacavir but not for tenofovir (56). Tenofovir in combination with emtricitabine and rilpivirine was used to treat patients in the present study except for patient number 4, who received abacavir in combination with lamivudine and dolutegravir. Probably, the potential stimulating effect of abacavir on CD11b surface expression was blocked by dolutegravir, which inhibits activation of nuclear factor (NF)-κB (57), an essential transcription factor for CD11b expression in neutrophils (58) and monocytes (59).

It was shown that MVA increases cell surface expression of CD86 in human monocyte-derived DCs (27). Here, we confirmed the upregulation of CD86 by MVA in DCs of HIV-ART patients, and the level of CD86 cell surface expression was even higher than in MVA-infected DCs of healthy persons. There is evidence that MVA increases the expression of CD86 in human monocyte-derived DCs mainly in non-infected bystander cells (60). Thus, we have to state that the effects of MVA on cell surface receptors as well as on chemokine expression that we observed cannot clearly be assigned to either infected cells or non-infected bystander cells. Consequently, that means that there could be a much higher number of cells affected by MVA than MVA-infected cells are virtually present in a system. This feature of MVA together with its ability to spread *in vivo* might have a systemic effect on the expression of cell surface receptors including CCR5.

Additionally, there is a consensus that cells of the monocyte/macrophage lineage are primarily infected by MVA *in vitro* and *in vivo* (29; 46; 61), and monocyte-derived tissue macrophages have a life span of months to years (62). Taken together, it could be possible that locally administered MVA modulates some systemic immune parameters for weeks or even months, which fits well in the concept of trained immunity (63; 64). In summary, here we found that MVA modulates the expression of many cell surface receptors, which can be different in healthy persons and HIV-ART patients in terms of quality and quantity. Moreover, we confirmed the ability of MVA to mature DCs and to induce chemokine expression in whole blood of HIV-ART patients and healthy persons. However, since many essential surface receptors were downregulated by MVA, it remains an open question whether the immunostimulatory activity of MVA is based only on the paracrine effects of MVA-induced cytokines or perhaps also on a not yet identified surface molecule, which is upregulated in MVA-infected cells.

## Data Availability Statement

Cytometric profiles collected in this study are available in the FlowRepository database under accession number FR-FCM-ZYVA.

## Ethics Statement

The studies involving human participants were reviewed and approved by the Comité de Protection des Personnes (CPP) Ile de France VII, under protocol number PP 14-003. The patients/participants provided their written informed consent to participate in this study.

## Author Contributions

AC, AL, and NT contributed to the conceptualization and methodology. AC, AL, EM, QJ, OL, RL, and NT contributed to the validation. AL, QJ, and NT contributed to the formal analysis. AC, AL, QJ, and NT contributed to the investigation. AL and OL contributed to the resources. AL, ML, and NT contributed to the writing of the original draft. AC and RL contributed to the acquisition of funding. AC, OL, RL, and NT contributed to the supervision. All authors contributed to the writing, reviewing, and editing.

## Conflict of Interest

The authors declare that the research was conducted in the absence of any commercial or financial relationships that could be construed as a potential conflict of interest.
