# Supplementary material for: Modulation of Cell Surface Receptor Expression by Modified Vaccinia Virus Ankara in Leukocytes of Healthy and HIV-Infected Individuals
Source: Front Immunol. 2020 Sep 8;11:2096. doi: 10.3389/fimmu.2020.02096 (PMC7506042; doi:10.3389/fimmu.2020.02096)
Supplement: Supplementary file 1 [file Data_Sheet_1.pdf]

Modulation of cell surface receptor expression by modified vaccinia virus Ankara in leukocytes of healthy and HIV-infected individuals

Supplementary materials

Adrien Leite Pereira[1], Quentin Jouhault[1], Ernesto Marcos Lopez[1], Antonio Cosma[1], Olivier Lambotte[1,2,3], Roger Le Grand[1], Michael H. Lehmann[4,*], Nicolas Tchitchek[1,*]

[1]CEA – Université Paris Sud 11 – INSERM U1184, Immunology of Viral Infections and Autoimmune Diseases, IDMIT Infrastructure, 92265 Fontenay-aux-Roses, France

[2]Inserm, U1184, Center for immunology of Viral Infections and Autoimmune Diseases, 94276 Le Kremlin-Bicêtre, France.

[3]APHP, Hôpitaux Universitaires Paris Saclay, Service de Médecine Interne-Immunologie Clinique, 94276 Le Kremlin-Bicêtre, France.

[4]Institute for Infectious Diseases and Zoonoses, Ludwig-Maximilians-Universität München, 80539 Munich, Germany

*** Correspondence:**
Nicolas Tchitchek
nicolas.tchitchek@sorbonne-universite.fr

Michael H. Lehmann
Michael.Lehmann@lmu.de
Orlataler@web.de

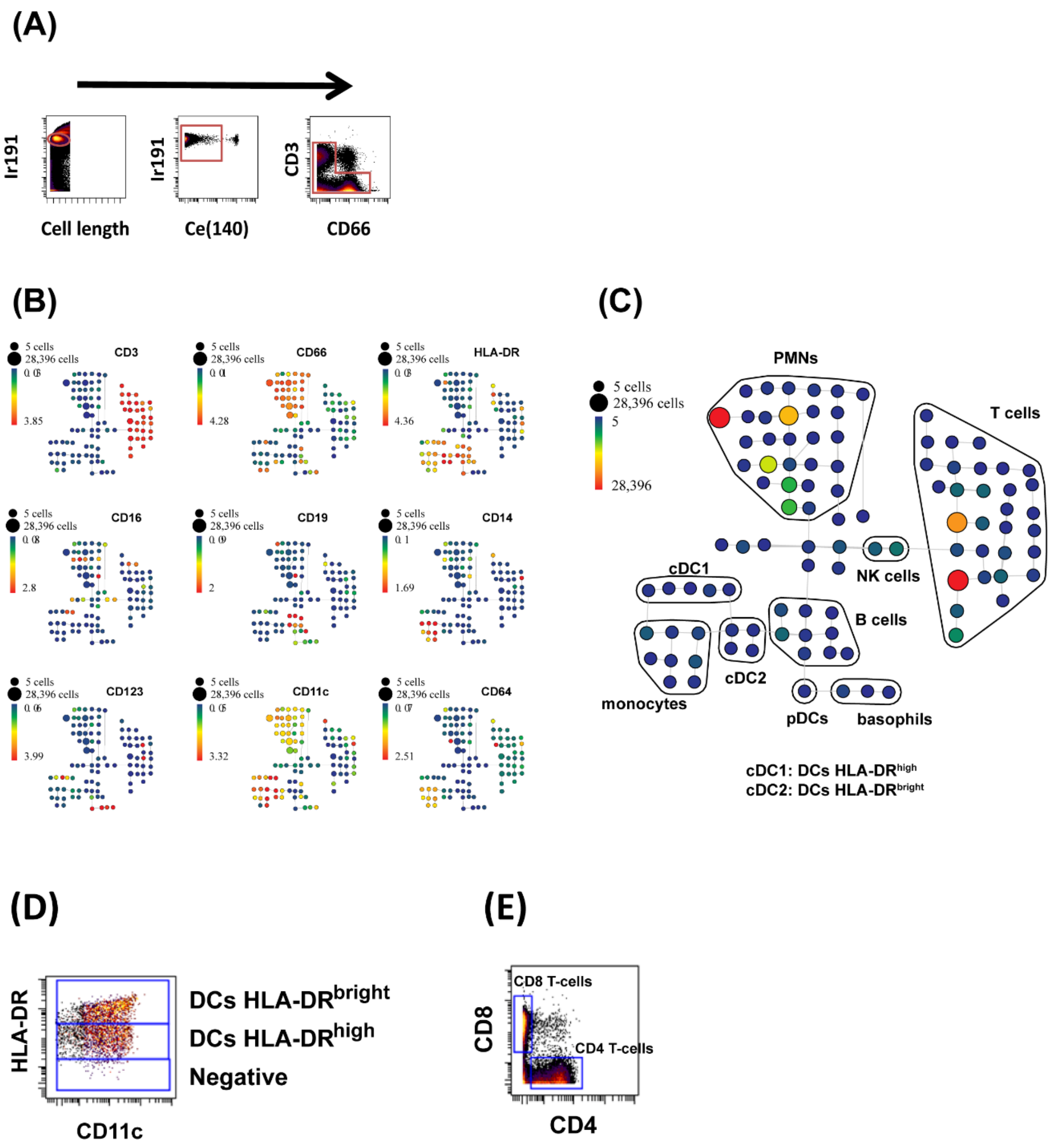

Supplementary Figure 1. Annotation of cell clusters identified by SPADE. (A) Samples were manually gated to exclude the EQTM Four-Element Calibration Beads, select singlets, and gate out nonspecific background generated by metal-conjugated Ab-binding eosinophils. (**B**) SPADE analysis was performed to identify 100 cell clusters using a 5% down-sampling parameter. Clustering was based on the levels of CD3, CD4, CD8, CD11c, CD14, CD16, CD19, CD32, CD64, CD66, CD123, HLA-DR, NKp80, and Perforin. The median expression of HLA-DR, CD3, CD11c, CD14, CD16, CD19, CD64, CD66, and CD123 were overlaid on the generated SPADE tree to annotate each cluster. **(C)** Eight leukocyte populations were identified based on the median expression of these specific markers. Granulocytes were designated as CD66^{+}, T-cells as CD3^{+}, NK cells as HLA-DR^{-} CD16^{+}, basophils as HLA-DR^{-} CD123^{+}, B-cell as HLA-DR^{+} CD19^{+}, monocytes as HLA-DR^{+} CD14$^{+/-}$ CD64^{+}, conventional dendritic cells (cDC) as HLA-DR^{+} CD11c+ CD14^{-} CD64^{-}, and plasmacytoid dendritic cells (pDC) as HLA-DR^{+} CD123^{+}. Thereafter, each of these eight cell populations was independently and computationally isolated. (**D**) Conventional dendritic cells were divided into two categories based on their HLA-DR levels: HLA-DR^{high} DCs and HLA-DR^{bright} DCs. (**E**) T-cells were divided into two categories based on their CD4/CD8 expression.

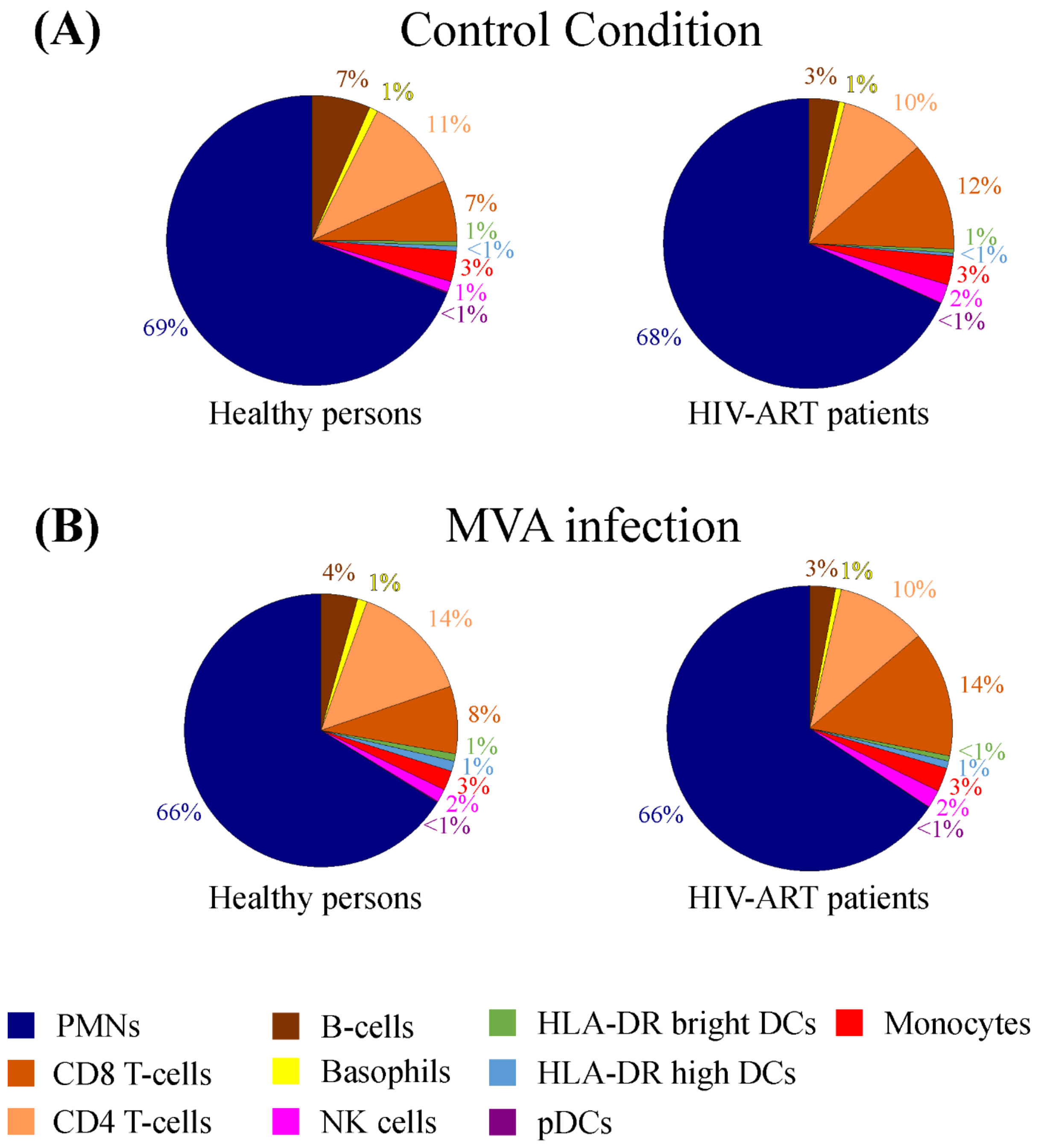

Supplementary Figure 2. Percentages of each cell type isolated from healthy persons and HIV-ART patients. Each pie chart represents the percentages of leukocyte populations isolated from the SPADE analysis for the whole set of healthy persons and HIV-ART patients in control condition (**A**) and after infection with MVA (**B**).
